# Comparative transcriptomic analysis of articular cartilage of post-traumatic osteoarthritis models

**DOI:** 10.1242/dmm.050583

**Published:** 2024-10-21

**Authors:** Sophie J. Gilbert, Jamie Soul, Yao Hao, Hua Lin, Katarzyna A. Piróg, Jonathan Coxhead, Krutik Patel, Matt J. Barter, David A. Young, Emma J. Blain

**Affiliations:** ^1^ Biomechanics and Bioengineering Research Centre Versus Arthritis, Biomedicine Division, School of Biosciences, The Sir Martin Evans Building, Cardiff University, Cardiff CF10 3AX, Wales, UK; ^2^ Biosciences Institute, Newcastle University, Centre for Life, Central Parkway, Newcastle upon Tyne NE1 3BZ, UK

**Keywords:** PTOA, ACL rupture, DMM, microRNA, Cartilage

## Abstract

Animal models of post-traumatic osteoarthritis (PTOA) recapitulate the pathological changes observed in human PTOA. Here, skeletally mature C57Bl6 mice were subjected to either rapid-onset non-surgical mechanical rupture of the anterior cruciate ligament (ACL) or to surgical destabilisation of the medial meniscus (DMM). Transcriptome profiling of micro-dissected cartilage at day 7 or day 42 following ACL or DMM procedure, respectively, showed that the two models were comparable and highly correlative. Gene ontology (GO) enrichment analysis identified similarly enriched pathways that were overrepresented by anabolic terms. To address the transcriptome changes more completely in the ACL model, we also performed small RNA sequencing, describing the first microRNA profile of this model. miR-199-5p was amongst the most abundant, yet differentially expressed, microRNAs, and its inhibition in primary human chondrocytes led to a transcriptome response that was comparable to that observed in both human ‘OA damaged vs intact cartilage’ and murine DMM cartilage datasets. We also experimentally verified *CELSR1*, *GIT1*, *ECE1* and *SOS2* as novel miR-199-5p targets. Together, these data support the use of the ACL rupture model as a non-invasive companion to the DMM model.

## Teaser

**Summary:** Pre-clinical studies of post-traumatic osteoarthritis are dominated by using the destabilisation of the medial meniscus (DMM) model. Here, we describe the transcriptomic similarity between the DMM mouse model and that for the more-rapid and reproducible mechanical rupture of the anterior cruciate ligament.

## INTRODUCTION

Post-traumatic osteoarthritis (PTOA), commonly referred to as secondary osteoarthritis (OA), arises following a known mechanical insult or traumatic injury and accounts for 12% of all patients presenting with OA (Brown et al., 2006). Traumatic destabilizing injury to the knee joint in young adults significantly increases the risk of developing OA in middle age (Gelber et al., 2000; Muthuri et al., 2011; Snoeker et al., 2020), particularly following a meniscal tear, intra-articular fracture and after cruciate ligament injury. Of patients with a diagnosed anterior cruciate ligament (ACL) or meniscus tear, ∼50% will develop pain and functional impairment of the joint 10–20 years post injury (Lohmander et al., 2007; Neuman et al., 2008). Epidemiological studies estimate an incidence of 77 in 10,000 patients reporting an acute knee trauma (Peat et al., 2014) and eight in 10,000 with ACL tears per annum (Sanders et al., 2016). These numbers will probably continue to increase due to a more active demographic, and is further compounded by the current lack of diagnosis following a traumatic injury and/or prognostic biomarkers to reliably predict whether OA will subsequently develop (Garriga et al., 2021). Therefore, understanding the aetiology of PTOA is imperative to define the early initiating events and identify effective diagnostics for subsequent treatment.

Several mechanically induced OA animal models have been established to recapitulate the pathological changes observed in human PTOA after an injury. The animal is subjected to a defined traumatic injury and temporal disease progression monitored to characterise the molecular, structural and functional outcomes. Traumatic injury to destabilise the joint is achieved following surgical transection or by application of a non-invasive mechanical load (comprehensively reviewed by Blaker et al., 2017; Christiansen et al., 2015; Narez et al., 2020). The most commonly used PTOA models include destabilisation of the medial meniscus (DMM) (Glasson et al., 2007) and non-surgical mechanically induced rupture of the ACL (Christiansen et al., 2015; Gilbert et al., 2018). Importantly, both models induce early inflammation, cartilage matrix loss resulting in fibrillation and destruction, synovitis, subchondral bone remodelling and formation of osteophytes (Burleigh et al., 2012; Gilbert et al., 2018; Lieberthal et al., 2015), all of which are clinical features that are also observed in human PTOA pathogenesis (Watt et al., 2016).

MicroRNAs (miRNAs) are small non-coding RNAs that regulate gene expression (Bartel, 2009). By using the DMM model, studies have previously characterised candidate miRNAs that are regulated during the early phases of OA pathogenesis (Kung et al., 2018, 2017b). Interestingly, transcriptomic analysis did not show an association between miRNA regulation and OA in the synovium (Kung et al., 2017b), subchondral bone (Kung et al., 2018) or serum (Kung et al., 2017a). However, in the articular cartilage, a subset of miRNAs is significantly regulated (Kung et al., 2018), and functional enrichment and data annotation analyses revealed responses to mechanical stimulation, apoptotic processes, and ECM structural and regulatory factors that are potentially involved in OA pathogenesis (Kung et al., 2018). miRNA analyses have also been reported in rat surgical ACL transection models with miR-27b (Zhang et al., 2020), miR-122 and miR-451 significantly elevated in the cartilage following joint destabilisation (Scott et al., 2021). To date, there have been no publications characterising the miRNA profile in cartilage harvested from the non-surgical load induced ACL rupture model, and only one mRNA transcriptome study has been published (Chang et al., 2017). Differential gene regulation has been observed for 1446 genes − including long non-coding RNAs (lncRNAs) − and, interestingly − compared to the mRNA profile of the DMM model − the greatest overlap observed was between ACL rupture at 1 week post injury and 4 weeks after DMM (Chang et al., 2017).

Therefore, in this study, we aimed to characterise the expression profiles of mRNA and miRNAs following mechanically induced ACL rupture to identify miRNAs as well as their downstream mRNA targets that are regulated during the early phase of PTOA disease progression. We also compared the miRNA gene signatures post ACL rupture with that in response to DMM and, of all miRNAs identified, found miR-199a-5p to be similarly differentially upregulated. Moreover, inhibition of miR-199a-5p in primary human chondrocytes revealed a role for this miRNA in extracellular matrix organisation.

## RESULTS

### Differential gene expression in response to abnormal mechanical loading of the joint

**Fig. 1.**
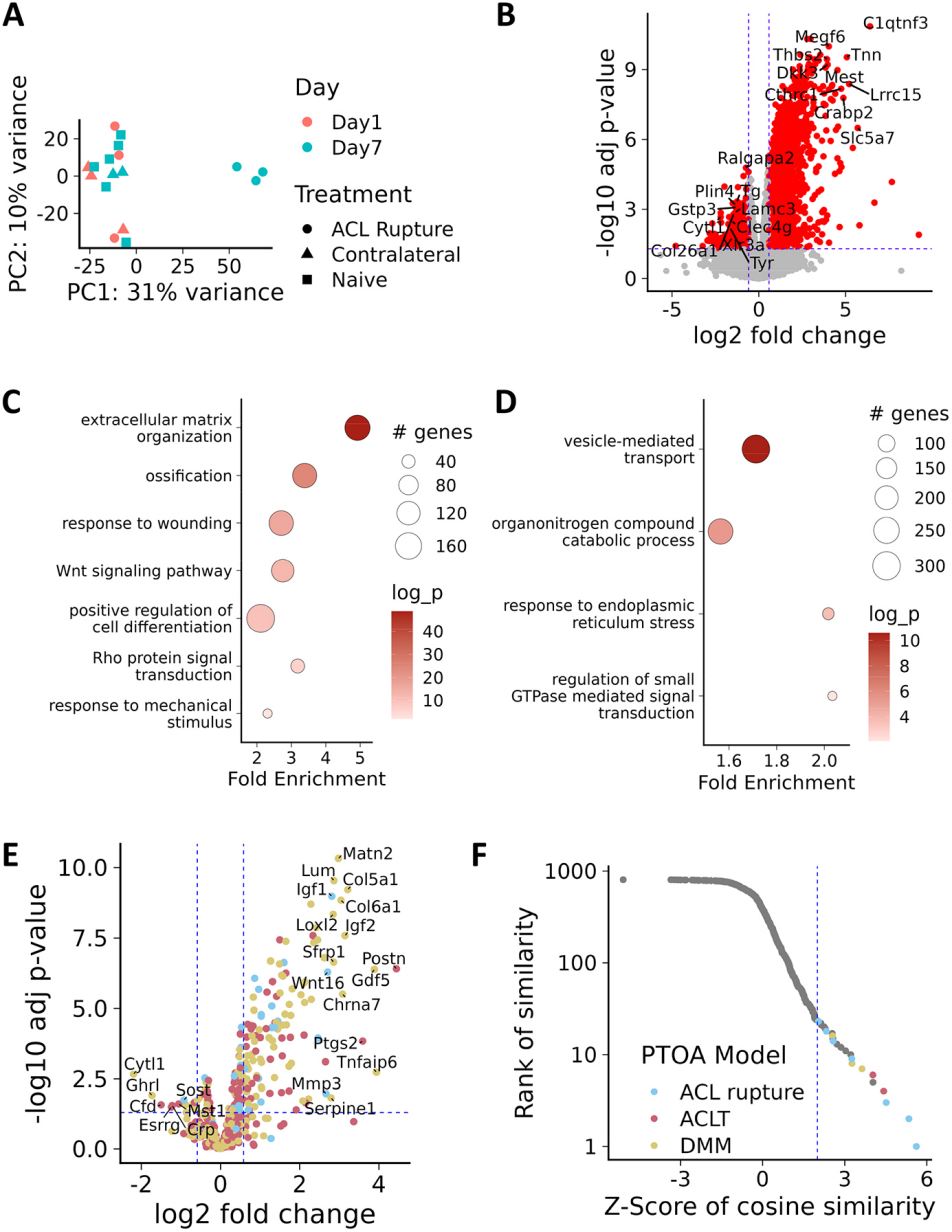
**Protective gene expression at early time points of abnormal mechanical loading following ACL rupture.** (A) Principal component analysis (PCA) plot of RNA-Seq data from ACL model of post-traumatic osteoarthritis (PTOA). PC1, principal component 1; PC2, principal component 2. (B) Volcano plot showing differential expression of all detected genes, red indicates genes that are significantly differentially expressed. Vertical dashed blue lines indicate 1.5-fold change, horizontal dashed blue line indicates the adjusted *P*-value (*P*<0.05). (C,D) Pathway enrichment plots of the enriched GO terms for significantly upregulated (C) and downregulated (D) genes. Shown are selected GO terms. Circle size indicates number of genes; colour intensity indicates the fold enrichment; log_10_ *P*-value (log_p). (E) Volcano plot showing the expression of known OA-affecting genes from day 7 ACL versus those of naïve control. Genes having detrimental, protective and ambiguous effects on OA are shown in yellow, red and blue, respectively. Vertical dashed blue lines indicate 1.5-fold change; horizontal dashed blue line indicates the adjusted *P*-value (*P*<0.05). (F) Graph showing the rank and value of the Cosine similarity z-scores for the comparison of day 7 ACL versus naïve control data with ∼800 datasets collated in SkeletalVis. The most similar profiles that correspond to PTOA models are highlighted and detailed in Table 1. Coloured dots indicate murine PTOA models as labelled.

Previously, we have reported a reliable and reproducible non-invasive loading model of joint injury with a defined point of injury, i.e. ACL rupture following mechanical insult (Gilbert et al., 2018). This model develops with early joint swelling accompanied by an acute inflammatory response, followed by joint degeneration and is histologically observable as early as 7 days post ACL rupture. To further characterise this model and define early gene expression changes, we performed an unbiased transcriptomics time course early after abnormal mechanical loading and ACL rupture on isolated cartilage from the femoral condyle. Joint injury samples at day 7 post injury were distinguishable from day 1 post-injury samples as well as from naïve control and contralateral control samples (Fig. 1A). We did not identify any gene as being significantly differentially expressed at day 1 post injury (versus naïve) but 2221 and 774 genes were significantly [≥1.5-fold, false discovery rate (FDR)≤0.05] up- and downregulated, respectively, at day 7 post injury (Fig. 1B, Table S3). Gene ontology (GO) enrichment analysis (Fig. 1C, Table S4) of the upregulated genes showed alterations in several anabolic terms including ‘extracellular matrix organization’ [adjusted (adj.) *P*=1.61E-49], ‘chondrocyte differentiation’ (adj. *P*=1.63E-16), ‘Wnt signaling’ (adj. *P*=3.042E-13), ‘Bmp signaling’ (adj. *P*=1.10E-10) as well as ‘response to mechanical stimulus' (adj. *P*=0.011). In general, GO pathways were less enriched for the downregulated genes but included GO terms related to molecule localisation or transport, e.g. ‘vesicle-mediated transport’ (adj. *P*=2.55E-11) along with GO pathways such as ‘response to endoplasmic reticulum stress’ (adj. *P*=0.0002) and ‘catabolic process’ (adj. *P*=1.10E-06) (Fig. 1D, Table S4).

**Table 1.**
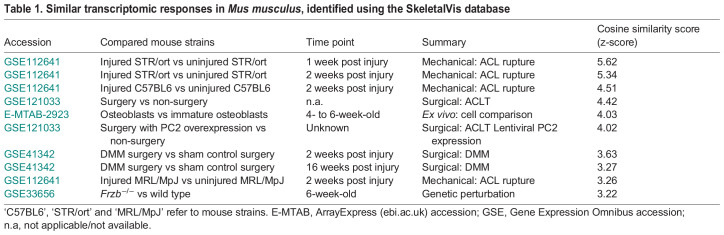
Similar transcriptomic responses in *Mus musculus*, identified using the SkeletalVis database

We have previously curated gene perturbations (genetic or pharmacological) by using animal models comprising joint damage (hereafter referred to as OATargets) (see Soul et al., 2021), and have categorised these genes into those having a ‘Protective’ (less damage with activation) or a ‘Detrimental’ (more damage with activation) effect. Amongst the genes upregulated with ACL rupture, those known to alter OA phenotypes when perturbed were primarily protective (68/217 Protective versus 36/199 Detrimental OATarget genes differentially expressed, *P*=0.002367). These included genes, such as *Matn2* (7.87 fold, adj. *P*=4.74E-11), *Sulf2* (5.49 fold, adj. *P*=3.68E-08), *Loxl2* (7.18 fold, adj. *P*=4.55E-09), *Gdf5* (14.80 fold, adj. *P*=4.11E-07) and *Sox9* (2.57 fold, adj. *P*=4.69E-05), which is suggestive of an anabolic transcriptomic response to the abnormal mechanical load (Fig. 1E). Upregulated known detrimental OATarget genes in the ACL model at day 7 included the protease *Htra1* (5.06 fold, adj. *P*=2.60E-08), *Adamts7* (3.14 fold, adj. *P*=5.58E-07), *Postn* (21.76 fold, adj. *P*=3.91E-07), *Acvr1* (2.49 fold, adj. *P*=1.12E-06), *Atf3* (2.59 fold, adj. *P*=3.23E-05) and the mechanical activated kinase *Fyn* (1.31 fold, adj. *P*=0.012). Comparable gene expression results were observed versus contralateral controls (Table S3).

To validate our transcriptomics data, we next compared the day 7 ACL rupture model gene expression responses with those of 800 transcriptional responses from skeletal cell types (Soul et al., 2019) listed in the SkeletalVis database. This analysis allowed assessment of the most similar gene expression responses (log2-fold changes) from a large database of potentially relevant datasets. Similarity between pairs of log2-fold changes were calculated using cosine similarity, where similar fold changes received positive scores and opposite fold changes received negative scores. These scores were then converted to z-scores (standard deviations from the mean similarity) to facilitate comparison across all datasets. Among the most similar were several other post-traumatic joint injury responses, including DMM and surgical transection of the ACL (ACLT) carried out at several time points, suggesting these generated data obtained from the abnormal loading model share common features with other models of PTOA (Fig. 1F, Table 1, Table S5). Interestingly, mouse knockout of the known chondrogenesis inhibitor *Frzb* was found to induce a similar transcriptomic response (Table 1). These data suggest a predominantly chondroprotective gene expression response in the joint shortly after abnormal mechanical loading.

**Fig. 2.**
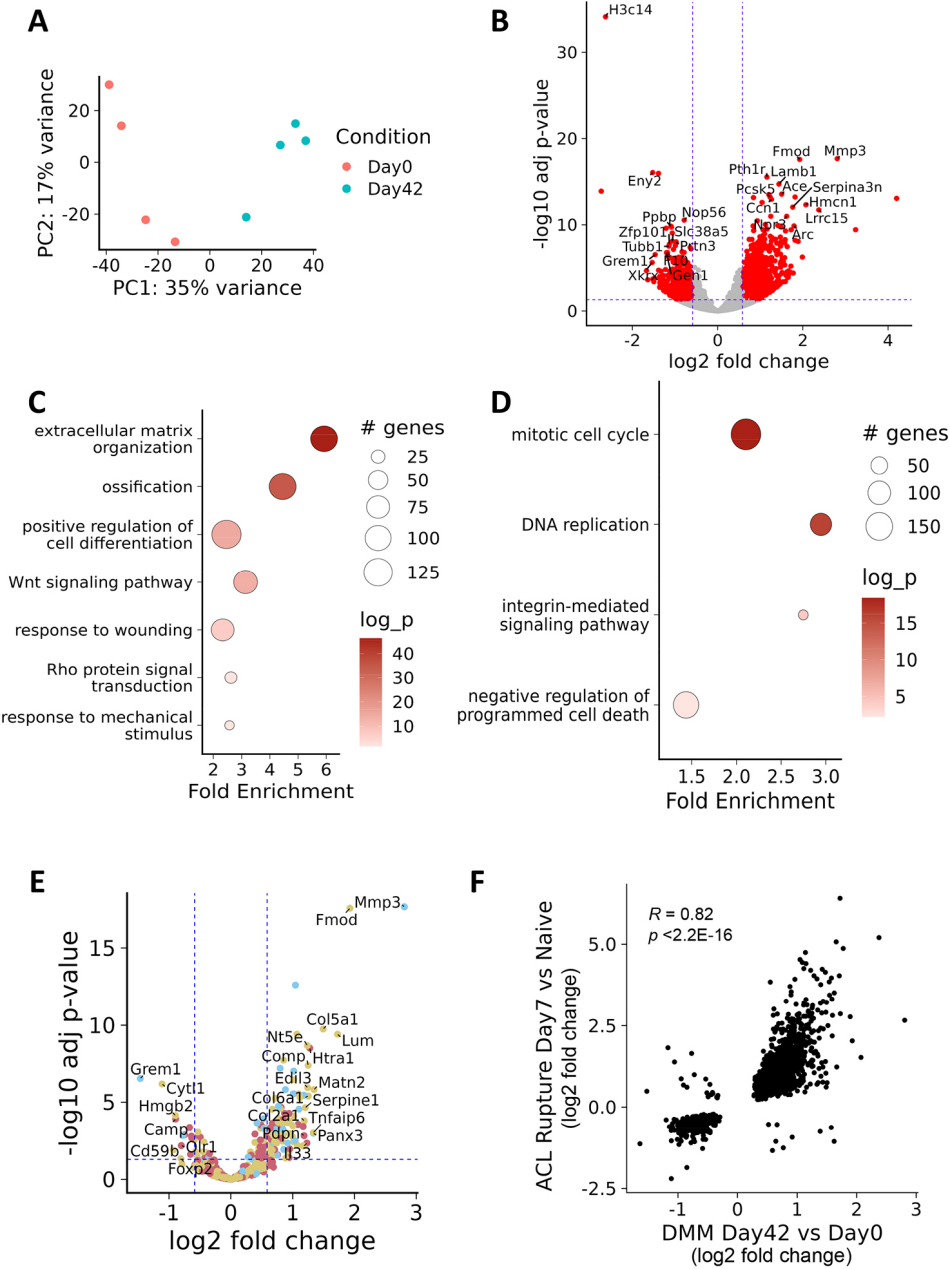
**Protective gene expression during DMM induction.** RNA-Seq data from the DMM model of post-traumatic osteoarthritis comparing gene expression of RNA from medial cartilage isolated at day 0 pre surgery, (red); naïve control animals versus animals 42 days post surgery (blue). (A) Principal component analysis (PCA) plot of animals (*n*=4 per condition). PC1, principal component 1; PC2, principal component 2. (B) Volcano plot showing differential expression of all detected genes, red dots indicate those significantly differentially expressed. Vertical dashed blue lines indicate 1.5-fold change, horizontal dashed blue line indicates the adjusted *P*-value (*P*<0.05). (C,D) Pathway enrichment plots of the enriched GO terms for significantly upregulated (C) and downregulated (D) genes following DMM OA induction. Shown are selected GO terms. Circle size indicates number of genes; colour intensity indicates fold enrichment −log_10_ *P* value (log_p). (E) Volcano plot showing the expression of known OA-affecting genes from day 42 DMM versus those of control (day 0). Genes having detrimental, protective and ambiguous effects on OA are shown in yellow, red and blue, respectively. Vertical dashed blue lines indicate 1.5-fold change, horizontal dashed blue line indicates the adjusted *P*-value (*P*<0.05). (F) Spearman correlation analysis of log2-fold expression of significantly differentially expressed genes (FDR <0.05) from ACL (day 7 ACL versus naïve control) and DMM (day 0 versus day 42) datasets.

Next, we directly assessed whether the gene expression changes seen in the ACL model were comparable to those at later time points within the DMM model, for which similar levels of joint degeneration have been observed (Gilbert et al., 2018; Glasson et al., 2007). We, therefore, performed RNA-Seq on RNA from medial knee cartilage caps dissected from individual mice pre- and day 42 post-DMM surgery. By principal component analysis (PCA) the different groups were clearly distinguishable (Fig. 2A). In all, we detected 2063 differentially expressed genes (≥1.5-fold, FDR≤0.05), of which 1167 were upregulated and 896 were downregulated (Fig. 2B, Table S6). GO enrichment analysis (Fig. 2C, Table S7) of the upregulated genes showed alterations in several anabolic terms including ‘extracellular matrix organisation’ (adj. *P*=5.78E-047) and ‘response to wounding’ (adj. *P*=1.5E-07). The downregulated genes were enriched in those within cell cycle terms (Fig. 2D, Table 7). Again, of the upregulated genes, those known to alter OA phenotypes when perturbed were mainly protective (53/200 Protective versus 22/181 Detrimental OATarget genes differentially expressed, *P*=0.00053) and similar to those described for the ACL-rupture model (Fig. 2E). Direct comparison of the differentially expressed genes between the ACL and DMM model showed a strong correlation (Spearman R=0.82, *P*<2.2E-16) (Fig. 2F), with <2% of differentially expressed genes being regulated in opposing directions, again indicating the shared transcriptomic responses in these PTOA models. None of the GO terms was enriched for these non-concordant genes.

### Analysis of differentially expressed miRNA

**Fig. 3.**
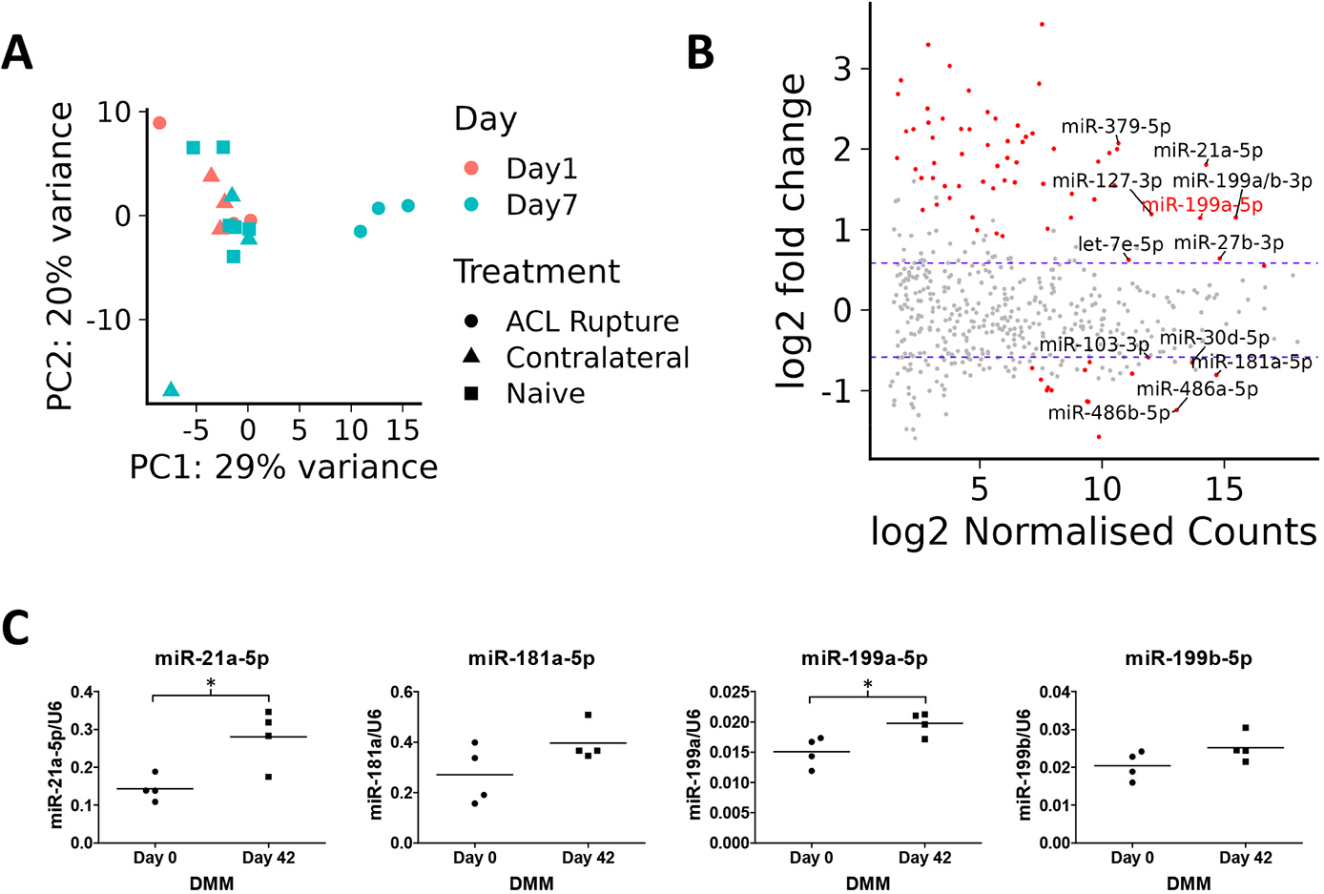
**Differential expression of miRNA upon abnormal mechanical loading.** (A) Principal component analysis plot showing of miRNA expression data from ACL model of post-traumatic osteoarthritis (PTOA). PC1, principal component 1; PC2, principal component 2. (B) MA plot, showing differential expression of most abundant miRNAs day 7 ACL versus naïve controls. Horizontal dashed blue lines indicate 1.5 fold change. Significantly differentially expressed miRNAs (FDR <0.05) are shown as red dots. miR-199a-5p is indicated in red. All non-significantly differentially expressed miRNAs are indicated by grey dots. (C) Selected miRNA expression in RNA from micro-dissected cartilage prior to DMM (circles, day 0) or 42 days post surgery (squares, day 42). Each data point represents a unique animal. Real-time PCR was performed in triplicates for each sample and normalised to the housekeeping *U6* small RNA using the calculation 2^−(ΔCt)^. Statistical comparisons were with an unpaired *t*-test with Welch‘s correction (*P<0.05).

Having characterised the mRNA expression profiles of the ACL rupture model, we next sought to identify potential post-transcriptional regulators of the observed differential expression. We performed small RNA sequencing to characterise the miRNA response to acute joint injury using the naïve, contralateral and ACL rupture femoral condyle cartilage cap samples described above. PCA, again, suggested that the naïve and contralateral limb have similar miRNA profiles, with only the ACL rupture model mice being distinguishable 7 days after mechanical insult (Fig. 3A). Similar to mRNA-sequencing results, no significant differential expression was observed day 1 post insult. Sixty-three statistically significant upregulated and 16 downregulated miRNAs were identified 7 days post mechanical loading (day 7 ACL versus naïve; Fig. 3B,  Table S8). Highly abundant, significantly differentially expressed miRNAs included miR-199a/b-3p (2.2 fold, adj. *P*=0.00285), miR-199a-5p (2.21 fold, adj. *P*=0.00144), and the previously reported mechanosensitive miRNAs miR-27b-3p (1.56 fold, adj. *P*=0.0316) and miR-21a-5p (3.5 fold, adj. *P*=0.000129) (Fig. 3B) (Stadnik et al., 2021). miR-199a-5p and miR-21a-5p were also found to be upregulated in cartilage following DMM surgery (Fig. 3C).

### Characterisation of miR-199-5p in human osteoarthritic chondrocytes

**Fig. 4.**
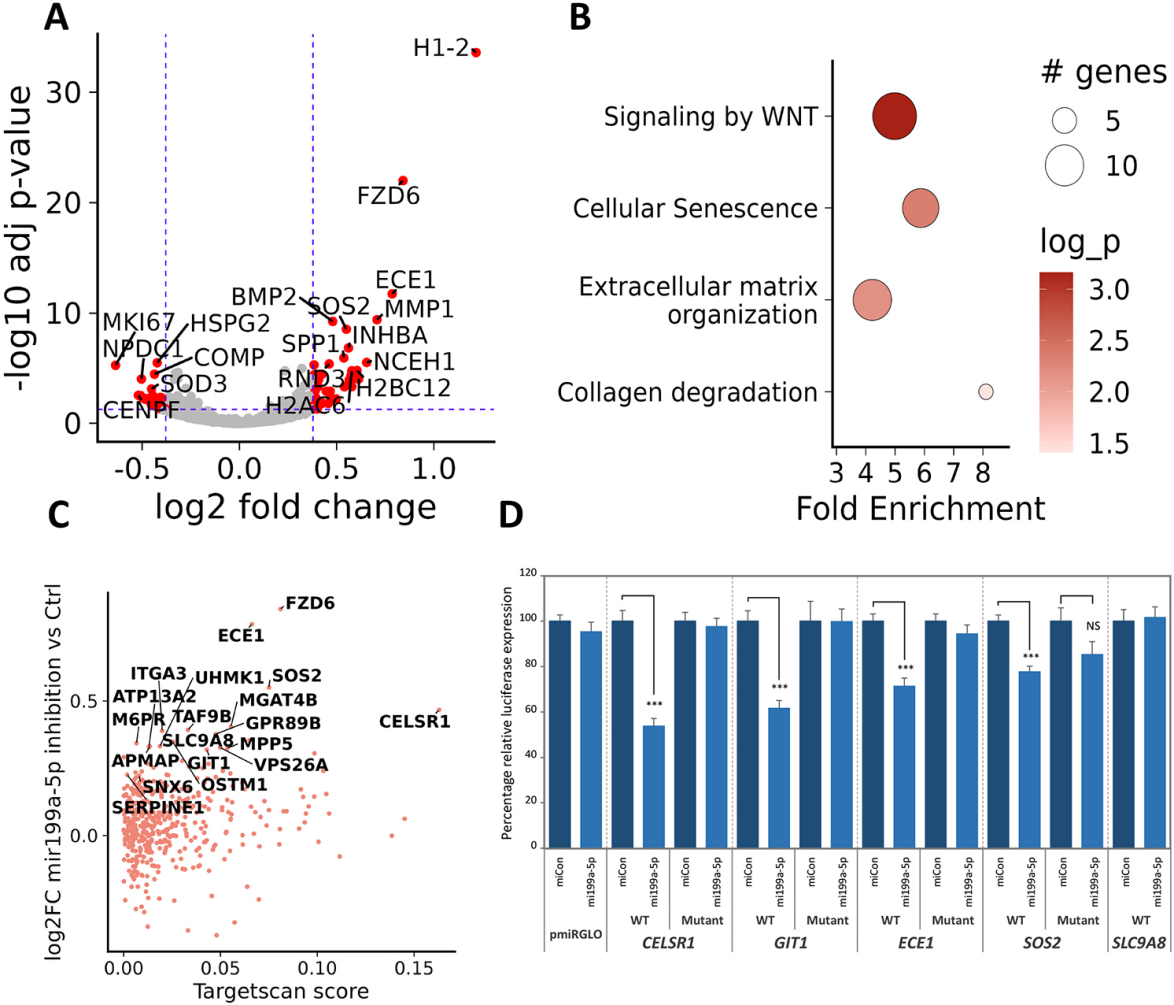
**Inhibition of miR-199a-5p induces a catabolic phenotype.** (A) Volcano plot showing the differential expression of mir-199a-5p inhibition in human OA chondrocytes (*n*=4 donors). Vertical dashed blue lines indicate 1.5-fold change, horizontal dashed blue line indicates the adjusted *P*-value (*P*<0.05). (B) Pathway enrichment plots of the enriched GO terms for genes significantly differentially expressed following inhibition of miR-199a-5p. Shown are selected GO terms. Circle size indicates the number of genes, the colour intensity indicates the fold-enrichment −log10 *P*-value (log_p). (C) TargetScan-predicted scores of mir199a-5p targets and their differential expression after inhibition of miR-199a-5p. (D) Testing of miR-199a-5p-predicted targets. The 3′UTRs of selected novel miR-199a-5p targets predicted by TargetScan were cloned into the reporter vector pmiRGLO, transfected into SW1353 chondrosarcoma cells with or without a mimic of miR-199a-5p (mi199a-5p, light blue) or non-targeting miR-mimic control (miCon, dark blue). After 24 h cells were lysed and Renilla and firefly luciferase levels determined, with the former being used to evaluate normalization of expression. Data shown are from four independent experiments performed six times and expressed relative (as a percentage) to the respective 3′UTR reporter co-transfected with control. Statistical comparisons were performed using two-tailed, unpaired Student's *t*-test (***P<0.001). NS, not significant.

Among the highly abundant differentially expressed miRNAs identified in both the ACL and DMM models, we have recently reported the role of miR-199a-5p as a positive regulator of human MSC chondrogenesis (Patel et al., 2023 preprint). Moreover, intra-articular delivery of this miRNA has also been shown to exert a significant protective effect in a rat OA model (Huang et al., 2023). Thus, we sought to further investigate the most-responsive potential target genes of miR-199a-5p in a human OA chondrocyte context. RNA-sequencing after inhibition of miR-199a-5p (Fig. S2) in primary human articular chondrocytes from four donors showed differential expression of 133 upregulated and 113 downregulated genes (Fig. 4A,  Table S9). Chondrocyte maturation associated genes, such as *MMP1* (1.63 fold, adj. *P*=3.85E-10), *MMP13* (1.28 fold, adj. *P*=0.0003), *BMP2* (1.4 fold, adj. *P*=5.5E-10), *INHBA* (activin-A) (1.48 fold, adj. *P*=1.4E-07), *SPP1* (Osteopontin) (1.45 fold, adj. *P*=11.1E-06) and *COMP* (−1.35 fold, adj. *P*=3.25E-05) were differentially expressed, suggesting a role for miR-199a-5p in regulating the chondrocyte phenotype. The host RNA for *MIR199A*, i.e. *DNM3OS*, was also upregulated (1.31 fold, adj. *P*=0.0024), suggesting some autoregulation. GO enrichment analysis showed significant enrichment of ‘extracellular matrix organisation’ (adj. *P*=0.00663) and ‘G1 DNA damage checkpoint’ (adj. *P*=0.0418) (Fig. 4B, Table S10). We also inhibited miR-199b-5p but were, however, unable to confirm selective miRNA inhibition (Fig. S2). Regardless, both miRNAs contain the same ‘seed sequence’ (nucleotides 2-8 located at the 5′ end) (Kozomara et al., 2019), so an unsurprisingly strong correlation of the fold changes (Spearman's correlation coefficient =0.89, *P*-value=2.2E-16) was observed (Table S9). Genes significantly upregulated after inhibition of miR-199b-5p – which were also elevated in the miR-199a-5p inhibition dataset – included the above-described *MMP1*, *MMP13* and *BMP2*.

Comparison of the gene expression response with other responses in SkeletalVis showed that miR-199a-5p inhibition resulted in a gene expression profile that was most similar to overexpression of the gene encoding transcription factor TBX5 (z-score=3.62). The gene expression response was also similar to human OA-damaged cartilage vs intact cartilage (z-score=3.43) and murine cartilage 6 weeks post DMM-surgery (WT DMM 6wkvsWT Sham 6wk) cartilage (z-score=3.19) − suggesting that miR-199a-5p depletion induced a gene expression profile similar to that seen in damaged OA cartilage (Table S1^1^).

Of the 133 genes for which expression significantly increased following inhibition of miR-199a-5p, 19 were TargetScan-predicted targets of miRNA (Fig. 4C). This is a significant (*P*=0.0004) enrichment, given there was only one predicted target within the 113 downregulated genes. We also explored comprehensive predictions from the microRNA Data Integration Portal (mirDIP) target prediction database (Tokar et al., 2018), which identified a further 14 miR-199a-5p-target genes within the list of 133 upregulated genes. The upregulated genes included experimentally validated targets of miR-199a-5p, such as *FZD6* (1.79 fold, adj. *P*=9.93E-23) (Kim et al., 2015) but also several less-well-described targets, such as *ECE1* (1.72 fold, adj. *P*=1.79E-12), *SOS2* (1.46 fold, adj. *P*=2.81E-09), *CELSR1* (1.38 fold, adj. *P*=0.0011), *GIT1* (1.25 fold, adj. *P*=0.0176) and *SLC9A8* (1.27 fold, adj. *P*=0.000242). Interestingly, of the predicted miR-199a-5p targets for which expression was increased upon inhibition of miRNA in OA chondrocytes, a significant proportion (*P*<0.02) was also increased in our murine ACL data. Finally, we tested whether these genes were direct targets of miR-199a-5p by using dual-luciferase reporter assays. 3′UTR-containing luciferase plasmids were co-transfected with control or mimics of miR-199a-5p into the SW1353 chondrosarcoma cell line. From these, *CELSR1*, *GIT1*, *ECE1* and *SOS2* were repressed by the miR-199a-5p mimic. This repression was lost by mutation of the predicted miRNA binding sequence within each 3′UTR (Fig. 4D).

## DISCUSSION

Our primary aim in this study was to define the early transcriptomic response of cartilage to mechanical damage in a non-surgical highly reproducible ACL rupture model. The non-surgical ACL rupture model transcriptomic signature was highly correlative with our own, and published, data from the well-established DMM OA model (Bateman et al., 2013; Loeser et al., 2013); this was despite the limitations of the study experimental design (Fig. S1) in that we did not assess the transcriptional aspect of the ACL model at an identical time point to the DMM model. The ACL rupture model is a rapidly progressing OA model and, at day 7, significant cartilage damage can be observed histologically. As, typically, cartilage has eroded completely by day 21 (Gilbert et al., 2018), the day 7 time point is more comparable and better reflects the extent of degeneration observed in the DMM model at day 42 (Glasson et al., 2007). Transcriptome assessment of early timepoints of the DMM model are confounded by the effects of surgery as determined through the use of sham surgeries (Bateman et al., 2013; Loeser et al., 2013). This highlights one benefit of the mechanically induced non-surgical ACL rupture model over that of DMM, in that it obviates the need for sham surgeries because the transcriptomic signature of the contralateral limb was equivalent to that of naïve animals, thus reducing animal numbers required to strengthen a study. The ACL model also better replicates a traumatic injury experienced by humans, allowing the study of early biological joint changes for the development of potential therapeutic interventions (Christiansen et al., 2015). The data presented here highlight the usefulness of the ACL rupture model as a non-invasive highly reproducible, alternative or companion to the DMM model. Both our DMM and, particularly, the ACL datasets clearly showed that the transcriptomic response to injury also involves repair activation. This included enrichment for GO pathways, such as ‘extracellular matrix organization’ and ‘cellular response to growth factor stimulus’. Upregulated genes in both datasets contained several collagen genes as well as the predicted proteases, especially those of the MMP family. In fact, others have reported that, following injury in a PTOA model, a distinct anabolic response, possibly mediated by the injured synovial tissue (Knights et al., 2023; Lai-Zhao et al., 2021).

We also performed small RNA sequencing (small RNA-Seq) following ACL rupture, defining miRNAs that are differentially and most abundantly expressed. These included miR-27b-3p, miR-21a-5p, and both the 5p and 3p arms from miR-199. Both miR-21 and miR-27 are known mechanosensitive miRNAs, and we have previously reported their regulation following the loading of *ex vivo* cartilage explants (Stadnik et al., 2021). In vivo, loss of miR-21a-5p alleviates cartilage matrix degradation in a murine model of temporomandibular joint OA (Zhang et al., 2020). However, whereas intra-articular injection of an miR-27-3p mimic (agomir) contributes to a synovial fibrotic response in murine knee OA, injection of an miR-27-3p inhibitor did not alter DMM-induced OA progression (Tavallaee et al., 2022).

Within the human genome, miRNA-199 is present at three locations − antisense within an intron of a dynamin (DNM) gene. The 3p arms of this miRNA are identical and generally those are most abundantly expressed, although the 5p arms are also readily detectible. The sequence of miR-199b-5p differs slightly from that of miR-199a-5p, although in all cases the ‘seed sequence’, i.e. nucleotides 2-8 located at the 5′ end, is identical (Kozomara et al., 2019). Mir-199a-2 is part of a miRNA cluster with miR-214 and miR-3120 and, although present within an intron of *DNM3*, the cluster is actually located within the long non-coding RNA (lncRNA) of dynamin 3 opposite strand (*DNM3OS*) on human chromosome 1q24 (Shepherdson et al., 2021). Patients with small deletions of this chromosomal region, affecting *DNM3OS* and the incumbent miRNAs, have skeletal abnormalities including short stature, microcephaly and brachydactyly (Lefroy et al., 2018), and, in mouse, are somewhat phenocopied by deletion of *Dnm3os* and, therefore, the miR199a∼214 cluster (Watanabe et al., 2008).

Several studies have addressed miR-199 in the context of cartilage or OA (Akhtar and Haqqi, 2012; Ali et al., 2020; Chao et al., 2020; Prasadam et al., 2016). In rodents with surgically induced OA, serum levels of miR-199a-5p increased (Lu et al., 2022), as we observed in cartilage following PTOA induction. The source of miR-199a-5p in serum is unclear but, in rats, inhibition of miR-199a-5p via intra-articular injection of an anti-miR (antagomir) following OA induction reportedly decreased joint inflammation and reduced cytokine levels (Lu et al., 2022). This observation is contradictory to the intra-articular injection of an miR-199a-5p agomir, which reduced cartilage damage in a rat PTOA model (Huang et al., 2023).

Given the conflicting literature surrounding miR-199a-5p, and since both physiological and supraphysiological overexpression of miRNA mimics can lead to spurious findings (Jin et al., 2015), we chose to manipulate the levels of the miRNA in cultured primary chondrocytes through inhibition with a specific hairpin inhibitor, Our RNA-Seq analysis revealed that inhibition of miR-199a/b-5p led to a transcriptome response similar to that of both human OA damaged versus intact cartilage and murine DMM cartilage 6 weeks post surgery. This included upregulation of the destructive collagenases *MMP1* and *MMP13*. Thus, we predict that the increase in miR-199a-5p observed following induction of PTOA *in vivo* is part of a chondroprotective response to the abnormal mechanical load experienced by the animal − in line with overexpression of miR-199a-5p *in vivo* reducing histological joint damage and expression of Mmp13 (Huang et al., 2023).

Overall, predicted targets of miR-199 were significantly enriched in the upregulated gene set following inhibition of miR-199a/b-5p. Amongst these targets was *FZD6*, a receptor involved in WNT signalling. *FZD6* has previously been reported as a miR-199a-5p target (Kim et al., 2015) but, interestingly, it is also a target of miR-140-5p, another important cartilage miRNA (Kim et al., 2015). Although WNT signalling has been extensively studied in cartilage development and OA (De Palma and Nalesso, 2021), little information pertains to a direct role for FZD6 in the tissue. Other direct targets of miR-199a-5p confirmed in this study include *GIT1*, *CELSR1*, *SOS2* and *ECE1*, the latter of which has previously been validated (Bao et al., 2018).

Several studies have profiled miRNAs in PTOA models. By using small RNA-Seq, Castanheira and colleagues (Castanheira et al., 2021) managed to identify just four miRNAs as being differentially expressed 8 weeks post DMM surgery. None of these miRNAs were significantly changed in our ACL rupture model, which may reflect the different models but is more likely to be due to different sampling, with the authors isolating RNA from whole mouse joints not just articular cartilage. Kung et al. (2018) also profiled miRNAs in dissected cartilage during DMM OA-induction. At 6 weeks post surgery, they identified 74 differentially expressed miRNAs, ten of which we also found differentially expressed in our 7-day post-ACL rupture data. However, only three miRNAs (miR-31-5p, miR486a-5p and miR-10a-5p) shared the same direction of gene expression change. The lack of correlation between our ACL and the published DMM miRNA datasets is surprising, especially given our finding that gene expression at day 7 post ACL and day 42 post DMM was highly correlative. Both experimental designs were similar, with approximately equal sample numbers and the use of pooled animals. The main difference is the technology used, i.e. small RNA-Seq in this study versus microarrays used by Kung et al. (2018). We did not perform small RNA-Seq following DMM, although it would have been valuable. However, in our DMM cartilage RNA, we were able to confirm upregulation of miR-199a-5p and miR-21a-5p by using real-time PCR, thereby validating our ACL miRNA profile and supporting a role for these miRNAs in cartilage tissue integrity.

In conclusion, we further characterised the ACL rupture model in this current study, providing − for the first time − both mRNA and miRNA-Seq data. The transcriptomic response of the DMM and ACL-rupture PTOA models was highly correlative. When adding the relative simplicity, speed and reproducibility of the ACL-rupture model, and that sham animals are not required, means it could be valuable in medium-throughput PTOA perturbation studies. The PTOA miRNA signature confirmed upregulation of numerous miRNAs, including those known to be regulated by altered biomechanical load. We also identified miR-199a-5p as being abundantly expressed and increased in both PTOA models. Inhibition of this miRNA in primary human chondrocytes resulted in a transcriptome profile that is similar to human OA damaged versus intact cartilage and murine DMM. Further work will be needed to resolve the role of miR-199 and its differing forms in OA *in vivo*.

## MATERIALS AND METHODS

### Animals

All animal experiments were performed under licences [Cardiff: P287E87DF, Newcastle: P8A8B649A] granted from the Home Office (UK) in accordance with the guidelines and regulations for the care and use of laboratory animals outlined by the Animals (Scientific Procedures) Act 1986 according to Directive 2010/63/EU of the European Parliament and conducted according to protocols approved by the Animal Ethics Committee of Newcastle University or Cardiff University and the Home Office, United Kingdom. Breeding and subsequent phenotyping was performed under licence P8A8B649A. All animal experiments were performed in compliance with the ARRIVE guidelines (Kilkenny et al., 2012). All animals were housed under 12:12 h light:dark photocycle, with food and water available *ad libitum.* A schematic of the experimental design and animals used is provided in Fig. S1.

### Mouse models of post-traumatic osteoarthritis

#### DMM mouse model

The DMM surgical model was performed essentially as described previously (Glasson et al., 2007). Briefly, eight C57Bl/6J male mice (25-30 g; bred in-house from Charles River, UK) were assigned to surgery or non-surgical groups at 11 weeks of age. Animals of the surgery group were given a pre-operative analgesic (buprenorphine), anaesthetised (isoflurane) and had their left knee medial meniscus destabilized by transecting the medial meniscus tibial ligament (MMTL) with a needle blade. The surgical wound was closed with 7-mm Reflex wound clips, which were removed 7 days post surgery. The day after surgery animals were given two doses of buprenorphine subcutaneously ∼8 h apart. Forty-two days post surgery, mice were euthanised by cervical dislocation.

#### ACL rupture mouse model

Twelve-week-old male C57Bl/6J mice (∼25 g; Envigo, Huntingdon, UK) were randomly assigned to either experimental or control groups, and randomly allocated to cages in groups of four or five. ACL rupture was performed as described previously (Gilbert et al., 2018). Briefly, mice were anaesthetized with isoflurane, and custom-built cups were used to hold the right ankle and knee in flexion with a 30° offset prior to the application of a 0.5-N pre-load (ElectroForce3200; TA Instruments, Elstree, UK). A single 12-N load at a velocity of 1.4 mm s^−1^ was then applied, resulting in ACL rupture; mechanical loading was always conducted in the morning. Buprenorphine (0.05 mg kg^−1^) was administered subcutaneously to mice at the start of the experiment; animals were able to move freely, and were monitored for welfare and lameness until termination of the experiment by cervical dislocation. Contralateral limbs, together with limbs from naïve mice served as controls (Gilbert et al., 2018). In total, 18 mice were subjected to ACL rupture (with six and 12 being euthanised at day 1 and day 7 post procedure, respectively). Six mice were naïve controls (Fig. S1).

### Total RNA extraction from mouse medial knee cartilage following DMM

Medial knee cartilage caps were dissected from individual mice (four per timepoint) pre- or 42 days post-PTOA induction. Tissue was washed three times with sterile Dulbecco's phosphate-buffered saline (DPBS) solution, placed in cryogenic vials and immediately frozen in liquid nitrogen. For grinding, tissue was placed in an autoclaved chamber with a ball (Retsch, Verder Scientific UK Ltd, UK) and 250 µl QIAzol lysis reagent (QIAGEN, Manchester, UK). The chambers were transferred to a Retsch MM200 mixer mill and tissue ground at vibration frequency of 25 Hz for 90 s. To this was added an additional 250 µl QIAzol lysis reagent, and the mixture was transferred to an RNase-free tube and incubated at room temperature for 5 min. Thereafter, 100 µl chloroform was added, and the sample was vortexed for 15 s, incubated at room temperature for 10 min followed by 5-min centrifugation (12,500 ***g***, at 4°C). The upper, RNA-containing, aqueous phase was then transferred into a new Eppendorf tube, and RNA and miRNA were purified using the mirVana™ miR Isolation Kit (Ambion, ThermoFisher Scientific, Loughborough, UK), followed by DNAse treatment (DNA-free™ DNA Removal Kit, Invitrogen, ThermoFisher Scientific) following the manufacturer's protocol.

### Total RNA extraction from mouse femoral condyle knee cartilage following ACL rupture

Femoral condyle cartilage caps were detached from underlying subchondral bone at the tidemark (Gilbert et al., 2018). Cartilage was pooled from mice as described (Fig. S1) from injured, uninjured (contralateral knees) or naïve limbs, and immediately snap frozen in liquid nitrogen prior to RNA extraction using TRIzol^®^ reagent according to manufacturer's protocol (ThermoFisher Scientific). Total RNA and miRNA were purified from the TRIzol^®^-cartilage mixture by using the mirVana™ miR Isolation Kit, followed by DNase treatment as described above and following the manufacturer's protocol. Samples were then assessed using a spectrophotometer (Nanodrop 1000, ThermoFisher Scientific) and 2100 Bioanalyzer (Agilent Technologies LDA UK Ltd, Stockport, UK) with A260/280 values between 1.8 and 2.0 and RNA integrity number (RIN) scores >8, respectively.

### Small RNA sequencing and small RNA analysis of murine knee cartilage

For DMM RNA-Seq, extracted RNA was DNase treated and RNA-Seq libraries were prepared using the Takara SMART-Seq v4 Ultra Low Input RNA kit, which incorporates rRNA depletion (Takara BIO Europe SAS, Saint-Germain-en-Laye, France). Libraries from four mice per timepoint were sequenced on an Illumina NovaSeq sequencer (Illumina, Cambridge, UK). For the ACL model, RNA-Seq sequencing libraries were prepared from the pooled samples (Fig. S1) using the TruSeq Stranded Total RNA with ribo-zero GOLD RNA library prep kit or the NEB Next Small RNA Library kit (New England Biolabs, UK) by Wales Gene Park (Cardiff, UK). The MiSeq Nano system (Illumina) was used to complete a sequencing library quality control, after which paired-end sequencing was performed using the Illumina Hi-Seq 2500 sequencer (Illumina). For both, total RNA-sequencing dataset data-quality control (QC) was via FastQC (v0.11.9) and reads were quality trimmed with Trimmomatic (0.39) (Bolger et al., 2014). Kallisto (v0.46.1) (Bray et al., 2016) was used for pseudo-alignment against mouse GRCm38 (release 103) transcriptome. Mapped transcript expression estimates were summarised to gene level using Tximport (v1.14.0) (Soneson et al., 2015). One ACL mRNA sample had a <50% RNA-Seq read mapping rate with Kallisto, so was removed from subsequent analysis.

For the small RNA-sequencing data of the ACL samples, the nf-core small RNA-Seq pipeline (revision 1.1.0) was ran with default parameters, except using the flags --genome GRCm38 --protocol ‘custom’ --three_prime_adapter AGATCGGAAGAGCACACGTCTGAACTCCAGTCAC --mirtrace_protocol illumina. Briefly, this pipeline performed quality control with fastQC (v0.11.9) and mirtrace (v0.11.9), trim galore (0.6.6) adaptor trimming and alignment against the mirbase mature miRNA sequence with bowtie1 (1.3.0) (Langmead et al., 2009). Aligned reads were counted with SAMtools (1.12) (Li et al., 2009).

PCA was performed using DESEqn (1.26.0) (Love et al., 2014) normalised and variance-stabilised gene expression data. For the DMM mouse model data, DESeq2 was used to calculate the log2-fold change (logFC) and *P*-values with the Wald test.

For both the ACL rupture model mRNA and miRNA count data, gene expression was normalised with edgeR. Limma-voom (v5.52.2) was used to calculate the log2-fold change (logFC) and *P*-values with moderated *t*-statistics (Ritchie et al., 2015). The duplicateCorrelation method in limma allowed accounting for the samples originating from the same mouse pool. *P*-values were corrected for multiple testing using the Benjamini−Hochberg method to provide the false discovery rate (FDR).

GO enrichment analysis was performed using GOseq (1.48.0) (Young et al., 2010) with up- and downregulated sets of significantly differentially expressed genes. GO terms with Benjamini–Hochberg (BH) corrected *P*-values of ≤0.05 were regarded as significant. To identify similar gene expression responses in existing musculoskeletal datasets, pre-processed fold changes were downloaded from the SkeletalVis (Soul et al., 2019) database and compared against the query fold-changes using cosine similarity. The cosine similarity score (interval between −1 and 1) is provided as the z-score [±standard deviation (±s.d.) of the mean] and allows comparison of transcriptional similarity relative to the background of skeletal cell type transcriptomic response.

To validate miRNA expression, RNA was reverse transcribed using the Applied Biosystems TaqMan^TM^ MicroRNA Reverse Transcription Kit (Life Technologies, Paisley, UK) and real-time RT-PCR was performed with specific TaqMan^TM^ MicroRNA assays (Life Technologies), and normalized to expression of the snRNA *RNU6B* (U6).

### Isolation of primary human articular chondrocytes, manipulation of miR-199a-5p levels and RNA-sequence analysis

Human articular chondrocyte (HAC) isolation from knee cartilage was performed as previously described (Barter et al., 2015). Tissue was donated by four patients (aged between 59 and 85; three female, one male; see Table S1) with diagnosed osteoarthritis and who were undergoing joint replacement surgery, with informed consent and ethics committee approval (REC 19/LO/0389). Briefly, macroscopically normal cartilage was removed from the subchondral bone and dissected into ∼1 mm pieces using scalpel and forceps. Enzymatic digestion was performed using hyaluronidase, trypsin and then collagenase overnight at 37°C (Cleaver et al., 2001). For modulation of miR-199 levels in HACs, Dharmacon miRIDIAN hairpin inhibitor against miR-199a-5p (catalogue no. IH-300607) or Dharmacon miRIDIAN miRNA Hairpin Inhibitor Negative Control #2 (catalogue no. IN-002005-01-05) were transfected into 40–50% confluent HACs using Dharmafect 1 lipid reagent (all Horizon Discovery, Cambridge, UK) at 100 nM final concentration. Twenty-four hours later, RNA was isolated following the miRVana protocol, quality assessed, and cDNA libraries were generated using the Illumina TruSeq Stranded mRNA protocol and sequenced on an Illumina NextSeq500 instrument. Kallisto, Tximport and principal component analysis (PCA) were used for analysis as described above, but with pseudo-alignment to the human GRCh38 (release 103) transcriptome. TargetScanHuman (release 8.0) (McGeary et al., 2019) and mirDIP (Tokar et al., 2018) were used to identify potentially direct targets of miR-199a/b-5p. DESeq2 (1.26.0) was used to calculate the log2-fold change (logFC) and *P*-values, calculated using the Wald test while accounting for the donor origin of each sample. Reactome pathway enrichment analysis [also known as gene set enrichment analysis (GSEA)] was performed by using GOseq (1.48.0) (Young et al., 2010) with either the upregulated or downregulated sets of differentially expressed genes. GO terms with Benjamini–Hochberg (BH) corrected *P*-values of ≤0.05 were regarded as significant. Data are available at NCBI GEO GSE229437.

### Cloning of plasmids and their transfection into SW1353 Cells

miRNA target 3′UTRs were amplified by PCR from human genomic DNA or synthesised as GeneArt DNA fragments (Life Techologies) to enable fusion using the In-Fusion HD cloning kit (Takara Bio). In-Fusion was carried out into the previously *Xho*I-linearized pmirGLO Dual-Luciferase miRNA Target Expression Vector (Promega, Southampton, UK), following the manufacturer's instructions (Table S2). Mutation of the miRNA seed within the plasmid was performed using the QuikChange II Site-Directed Mutagenesis Kit (Agilent Technologies) or by altering the GeneArt DNA fragment sequence synthesized (Table S2). All vectors were sequence verified. SW1353 chondrosarcoma cells were cultured in 96-well plates overnight to 50% confluence (1.5×10^4^ cells/cm^3^). Cells were first transfected with 3′UTR luciferase constructs (10 ng) by using the FuGENE HD transfection reagent (Promega) for 4 h, and then transfected using Dharmafect 1 with Dharmacon miR-199a-5p mimic (50 nM) or miRNA mimic non-targeting control #2 (Horizon Discovery, Cambridge, UK). Twenty-four hours post the second transfection, cell lysates were assayed for levels of Firefly and Renilla luciferase by using the Promega Dual-Luciferase Reporter Assay System measured on a GloMax 96 Microplate Luminometer (Promega). Statistical comparisons were performed using two-tailed, unpaired Student's *t*-test.

### Code availability

Code to generate the bioinformatics figures is available at https://github.com/soulj/OAModelmicroRNA

## Supplementary Material

10.1242/dmm.050583_sup1 Supplementary information

Table S1. Patient demographics for the HAC isolation

Table S2. Oligonucleotide sequences

Table S3. ACL gene expression data

Table S4. ACL data GO term analysis

Table S5. ACL data comparison with SkeletalVis

Table S6. DMM gene expression data

Table S7. DMM data GO term analysis

Table S8. ACL microRNA expression data

Table S9. miR-199-5p inhibition in HAC gene expression data

Table S10. miR-199-5p inhibition in HAC data GO term analysis

Table S11. miR-199-5p inhibition in HAC data comparison with SkeletalVis
