# Supplementary material for: Comparative transcriptomic analysis of articular cartilage of post-traumatic osteoarthritis models
Source: Dis Model Mech. 2024 Oct 21;17(10):dmm050583. doi: 10.1242/dmm.050583 (PMC11524441; doi:10.1242/dmm.050583)
Supplement: Supplementary information [file dmm-17-050583-s1.pdf]

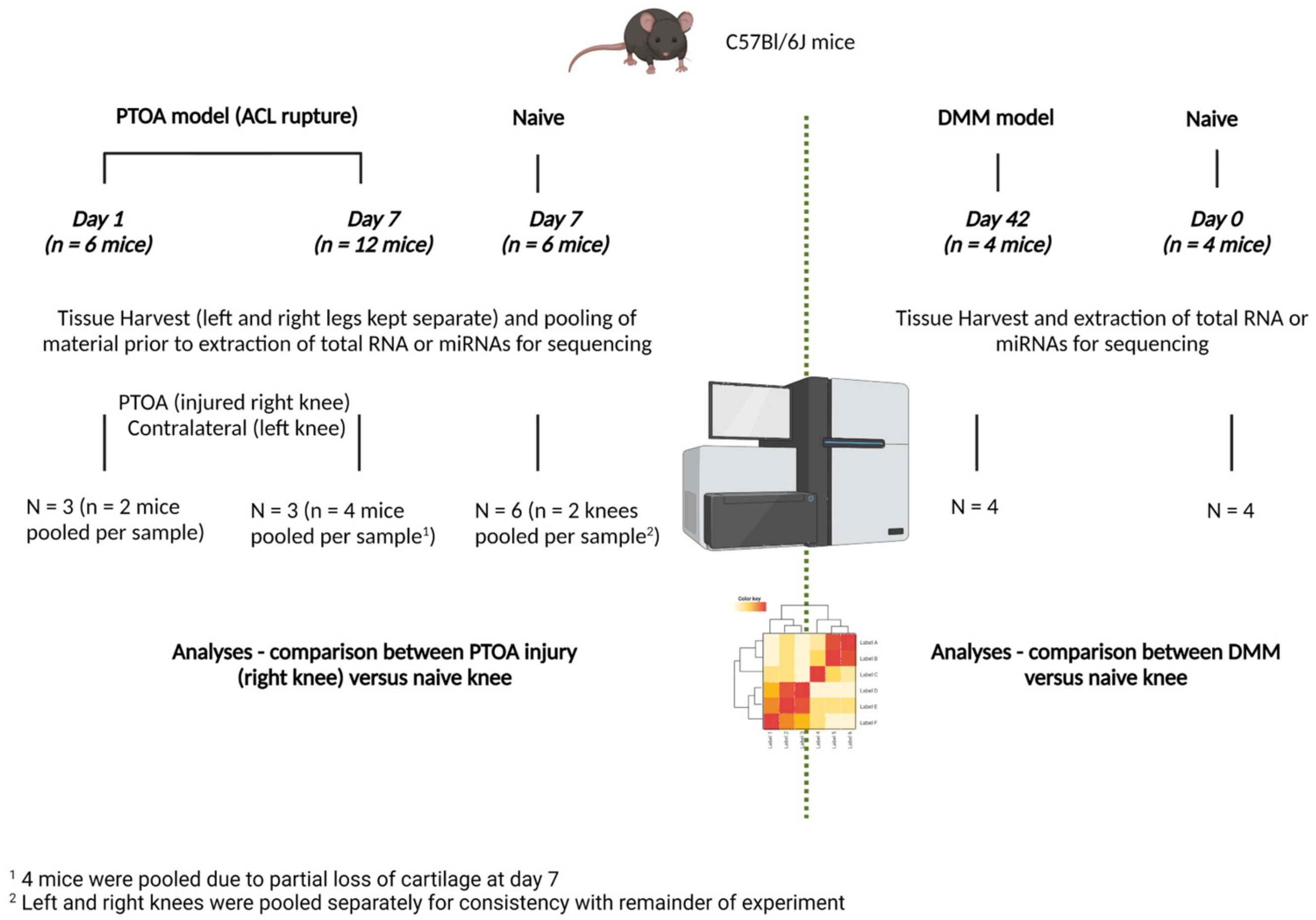

Fig. S1. Schematic of the experimental design and samples/animals used for RNA-seq

Disease Models & Mechanisms • Supplementary information

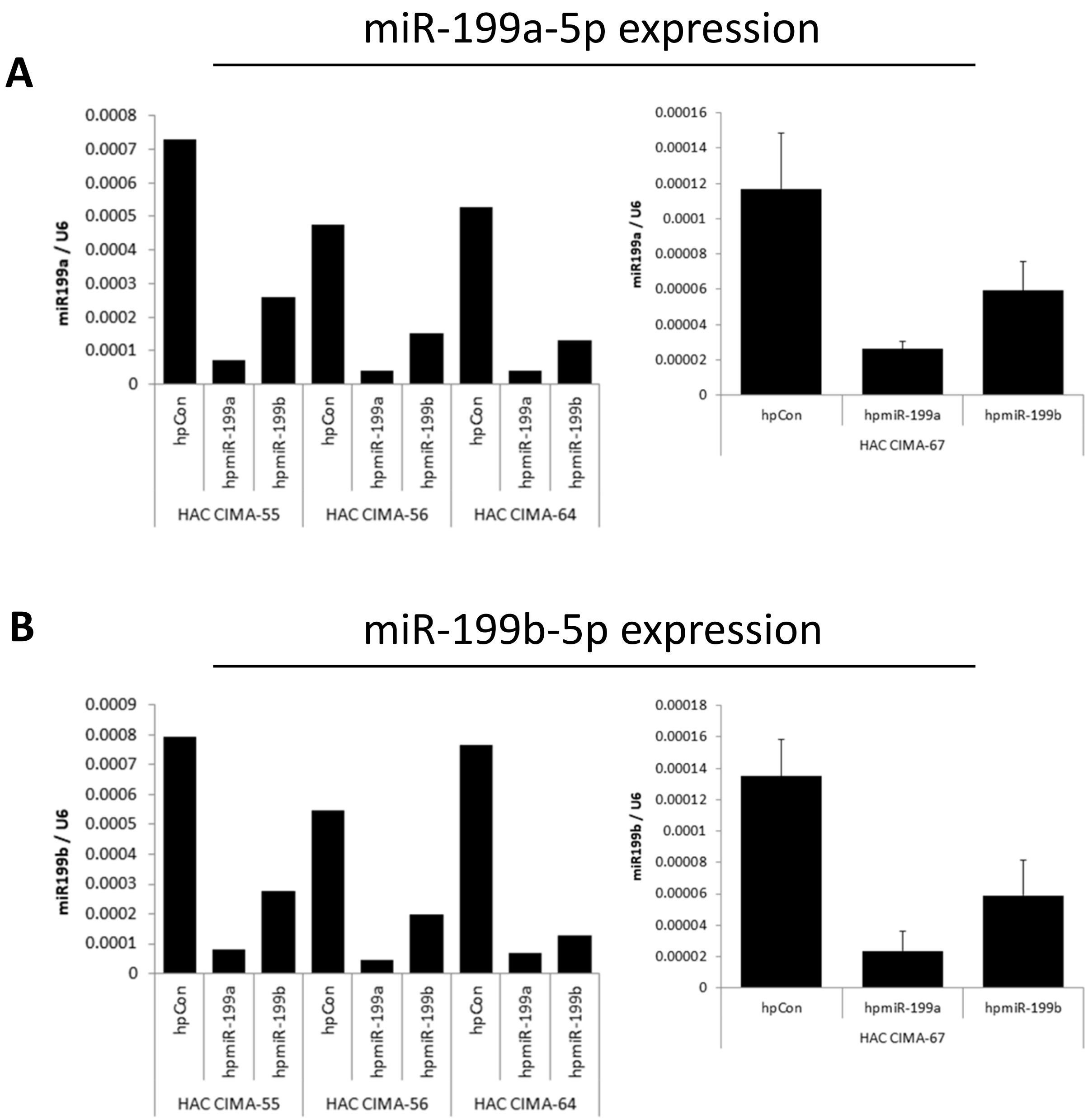

Fig. S2. Confirmation of inhibition of miR-199a-5p or miR-199b-5p in primary human articular chondrocytes used for RNA-seq

qRT-PCR measurement of miR-199a-5p (A) and miR-199b-3p (B) in four primary human articular chondrocyte donors (HAC), CIMA-55, -56, -64 and -67, following inhibition of the miRNA with the indicated control or targeting hairpin inhibitor. Note, the experiment for donor CIMA-67 was performed in triplicate. All data were normalised to the housekeeping short RNA U6. The hairpin inhibitors successfully inhibited the target miRNA but we were unable confirm isoform specificity. This could be because the either the inhibitors of the RT-qPCR assays cannot distinguish between miR-199a or b-5p. CIMA refers to the anonymous patient study ID.

Table S1. Patient demographics for the HAC isolation

Available for download at
https://journals.biologists.com/dmm/article-lookup/doi/10.1242/dmm.050583#supplementary-data

Table S2. Oligonucleotide sequences

Available for download at
https://journals.biologists.com/dmm/article-lookup/doi/10.1242/dmm.050583#supplementary-data

Table S3. ACL gene expression data

Available for download at
https://journals.biologists.com/dmm/article-lookup/doi/10.1242/dmm.050583#supplementary-data

Table S4. ACL data GO term analysis

Available for download at
https://journals.biologists.com/dmm/article-lookup/doi/10.1242/dmm.050583#supplementary-data

Table S5. ACL data comparison with SkeletalVis

Available for download at
https://journals.biologists.com/dmm/article-lookup/doi/10.1242/dmm.050583#supplementary-data

Table S6. DMM gene expression data

Available for download at
https://journals.biologists.com/dmm/article-lookup/doi/10.1242/dmm.050583#supplementary-data

Table S7. DMM data GO term analysis

Available for download at
https://journals.biologists.com/dmm/article-lookup/doi/10.1242/dmm.050583#supplementary-data

Table S8. ACL microRNA expression data

Available for download at
https://journals.biologists.com/dmm/article-lookup/doi/10.1242/dmm.050583#supplementary-data

Table S9. miR-199-5p inhibition in HAC gene expression data

Available for download at
https://journals.biologists.com/dmm/article-lookup/doi/10.1242/dmm.050583#supplementary-data

Table S10. miR-199-5p inhibition in HAC data GO term analysis

Available for download at
https://journals.biologists.com/dmm/article-lookup/doi/10.1242/dmm.050583#supplementary-data

Table S11. miR-199-5p inhibition in HAC data comparison with SkeletalVis

Available for download at
https://journals.biologists.com/dmm/article-lookup/doi/10.1242/dmm.050583#supplementary-data
